# Clinical importance of the relationship between parietal foramen variations and the sagittal suture

**DOI:** 10.1097/MD.0000000000042075

**Published:** 2025-04-04

**Authors:** Nazire Kilic Safak, Zekiye Karaca Bozdag, Ayca Pamukcu, Ozkan Oguz

**Affiliations:** a Department of Anatomy, Faculty of Medicine, Cukurova University, Adana, Turkey; b Department of Anatomy, Faculty of Medicine, Istanbul Yeni Yuzyil University, Istanbul, Turkey; c Department of Business Management, Land Forces, Vocational High School of Non-commissioned Officer, Milli Savunma University, Balikesir, Turkey.

**Keywords:** morphometry, parietal foramen, sagittal suture

## Abstract

This study aims to determine the prevalence, number, and localization of the parietal foramen in relation to the sagittal suture. Forty skulls (80 parietal bones), whose age and sex were unknown, from the Department of Anatomy, Faculty of Medicine, Cukurova University, were studied. Morphometric measurements were performed using a digital caliper. The presence of the parietal foramen and its number, localization, and distance from the sagittal suture were evaluated in this study. SPSS v.20 software was used for statistical analysis. In addition to descriptive statistical methods, the Mann–Whitney U-test was used to evaluate the quantitative data. The parietal foramen was not detected in 19 skulls (47.5%) but was observed unilaterally in 10 skulls (25%) and bilaterally in 11 skulls (27.5%). The parietal foramen was detected in 32 parietal bones (40%). Furthermore, it was observed as single in 30 parietal bones (37.5%) and as double in 2 parietal bones (2.5%); however, the triple parietal foramen was not detected. There were no double foramens in the right parietal bone. The foramen was not observed in 48 (60%) of the parietal bones. The mean distance between the parietal foramen and sagittal suture was as 7.42 ± 5.64 mm. No statistically significant differences were detected between the parietal foramina in terms of their distances to the sagittal suture on either the right or left side (*P* > .05). Knowledge of the localization of the parietal foramen and its relationship with the sagittal suture helps understand the relationship between the dural venous sinuses and scalp veins. Furthermore, an understanding of these variations may aid in the detection of congenital anomalies. We believe that the results of this study will contribute important morphometric data for anatomists and clinicians.

## 1. Introduction

Parietal bones, which have a complex vascular network in modern humans, constitute the vault and the lateral sides of the skull.^[1,2]^ Furthermore, the parietal foramen is usually located near the sagittal suture at the posterior aspect of the parietal bones, and its presence is inconsistent. The locations, dimensions, numbers, and shapes of these small openings are highly variable.^[3,4]^ In fact, variations are very common, and sometimes the description of a normal structure is much more difficult; thus, knowledge of the differences is vital.^[5]^

The skull is the most complex bone in the body, covering crucial brain structures.^[6]^ The foramina of the skull have fascinated anatomists and anthropologists from the past to the present.^[3,7]^ The parietal foramen connects the middle meningeal and scalp arteries and augments vascularization of the galea.^[8]^ On the other hand, it involves the emissary veins, which connect the scalp veins with the superior sagittal suture.^[9]^ Consequently, in addition to enlightening neurovascular anatomy, having sufficient data about these variations could help prevent clinical complications.^[10,11]^ Even though it is highly important, there are a limited number of studies regarding the location, number, and variations of the parietal foramen.^[3,12]^ The purpose of the current study was to determine the prevalence, number, and localization of the foramen parietale according to sagittal suture.

## 2. Materials and methods

**Figure 1.**
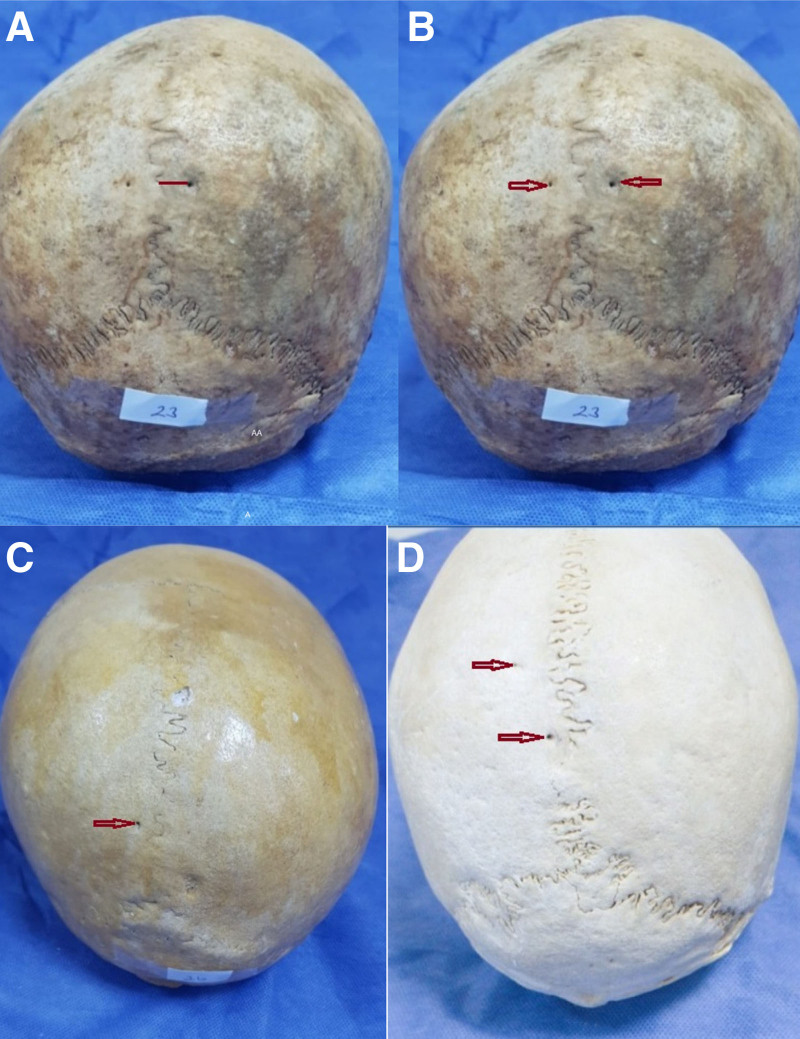
Parietal foramen: (A) measurement of the parietal foramen-sagittal suture distance, (B) bilateral on skull, (C) left unilateral on skull, and (D) left parietal bone with double parietal foramen.

In this study, 40 unknown skulls (80 parietal bones), whose age and sex were unknown, from the Department of Anatomy, Faculty of Medicine, Cukurova University, were used. Skulls that were deformed, fractured, or showed pathological changes were excluded from this study. First, the presence, number, and location of the parietal foramen were noted, and patency was checked using a small needle. Subsequently, the shortest vertical distance between the sagittal suture and parietal foramen was measured using a digital caliper to the nearest 0.1 mm (Fig. 1A). All the measurements were performed by the same investigator (NKŞ). Ethical approval was obtained from the Cukurova University Non-Interventional Clinical Research Ethics Committee for the study (2024, No: 141).

Statistical analyses were performed using the SPSS software (version 20.0; IBM Corp., Armonk, NY). The Shapiro–Wilk test and histograms were used to assess the normality of the variables. In addition to descriptive statistical methods (i.e., mean, standard deviation, and percentage), the Mann–Whitney *U* test was used to evaluate any quantitative data that did not fit the normal distribution. The significance level was set at *P* < .05.

## 3. Results

**Figure 2.**
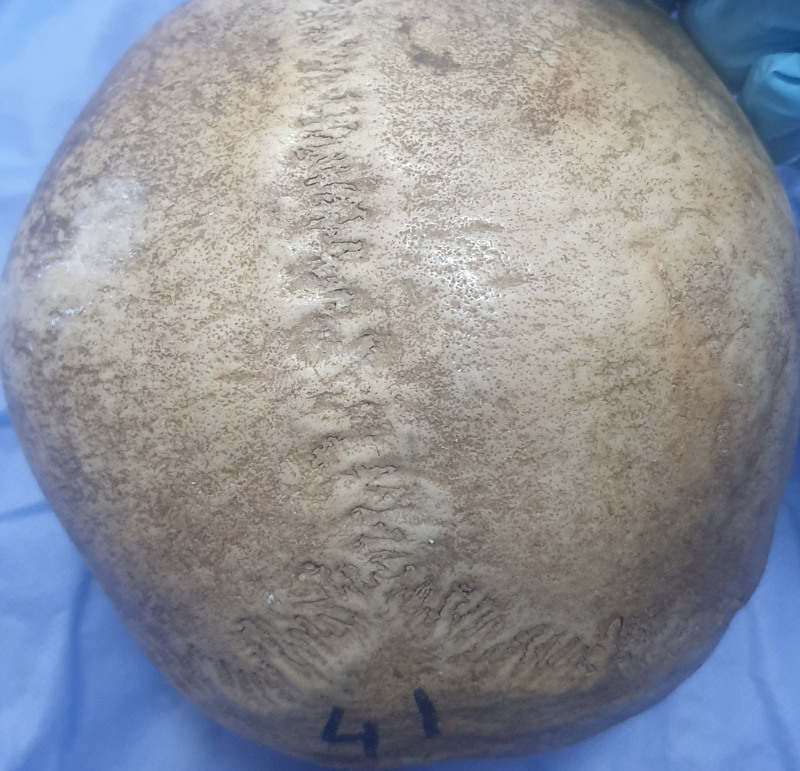
Absent parietal foramen.

**Figure 3.**
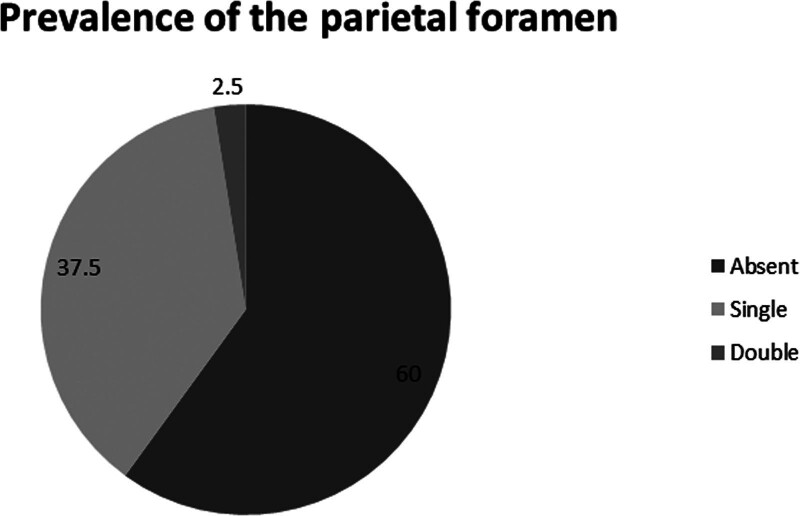
Prevalence of the parietal foramen on parietal bones (n = 80).

The parietal foramen was not detected in 19 skulls (47.5%) but was observed unilaterally in 10 skulls (25%) (Fig. 1C and D) and bilaterally in 11 skulls (27.5%) (Fig. 1B). In addition, the parietal foramen was detected in 32 (40%) parietal bones. Furthermore, it was observed as single in 30 parietal bones (37.5%) and double in 2 parietal bones (2.5%), but the triple parietal foramen was not detected in any of the parietal bones. There was no double foramen in the right parietal bone. The foramen was not observed in 48 (60%) of the parietal bones (Figs. 2 and 3). The mean values of the distance of the parietal foramen to the sagittal suture were found as 7.42 ± 5.64 mm in total, 6.75 ± 2.17 mm on the right side and 8.01 ± 7.55 mm on the left side. No statistically significant differences were detected among the parietal foramina in terms of their distances to the sagittal suture on the right and left sides (*P* > .05) (Table 1).

**Table 1:** Distance of the parietal foramen to the sagittal suture.

Parietal foramen distance (mm)	N	Mean	Standard deviation
Right	16	6.75	2.17
Left	18	8.01	7.55
Total	34	7.42	5.64

## 4. Discussion

In this study, the incidence of parietal foramen was detected in 21 skulls (52.5%). And bilaterally parietal foramen was observed higher than unilaterally parietal foramen. No statistically significant differences were detected in terms of parietal foramen-sagittal suture distance between 2 sides (*P* > .05). Most of the skull bones originate from intramembranous ossification, similar to the 2 parietal bones. Parietal bones begin to develop in the 8th week postconception.^[13,14]^ Parietal bones are quadrilateral and consist of lateral sides and the roof of the skull. Delayed or slow ossification in the posterior third of the parietal bone may result in a V-shaped notch, pars obelica, and sagittal and third fontanels.^[9]^ The parietal foramen is derived from ossification when the closure of the third fontanel occurs.^[12]^ The parietal foramen provides a connection between the dural venous sinuses and scalp veins.^[3,4]^ Some studies that have focused on enlarged parietal foramina have aimed to clarify the reasons. It has been claimed that insufficient ossification might result in an enlarged parietal foramen.^[15]^ In a case report, Baydin reported an association between the parietal foramen and headache.^[16]^ An investigation of meningeal anastomosis in the parietal foramen in cadaveric samples indicated that the frequency of the parietal foramen was 50%.^[8]^ A study on the relationship between suture complexity and parietal foramen indicated that the sagittal suture was less complex near the parietal foramen.^[12]^ A relationship was also reported between the skull capacity and surface area of the left and right sides of the parietal foramina.^[17]^ An investigation regarding variations in the size and symmetry of the skull foramina found that the parietal foramen was bilateral in 40% of skulls, unilateral in 30%, and absent in 20%. These results were higher in terms of the presence of the parietal foramen than those in the current study. As their study included skulls with foramina, we believe that this difference depended on the study sample of that particular study.^[18]^ The prevalence of the parietal foramen ranged from 22.2% to 62.7% according to the study population in research conducted in different populations. The study results herein were more similar to those of the Modern Palestine population when compared to another study.^[5]^

The frequency of the parietal foramen was observed unilaterally in 29% of skulls and bilaterally in 15% in a study of variations in 103 dried human skulls in an Indian population.^[6]^ In another study, the presence of the parietal foramen was observed in 77.08% of patients in India. Additionally, the parietal foramen was observed bilaterally in 62.5% of skulls and unilaterally in 29.2%. In addition, their study mentioned that the location of the parietal foramen was mostly between the posterior and middle thirds of the parietal bones.^[9]^ An anatomical study by Halagatti & Sagar in India showed that the prevalence of the presence of parietal foramen was 75.6%, and out of 430 parietal bones, a single foramen was found in 283 bones, double foramen was found in 35 bones, and triple foramen was found in 7 bones. They indicated that the mean distance between the parietal foramen and the sagittal suture was 6.4 ± 2.5 mm on the right side and 6.6 ± 2.6 on the left side.^[3]^ Murlimanju et al conducted a study in India and emphasized that the incidence of parietal foramen involvement was 71.5%. Of the 116 parietal bones, the incidence of unilateral parietal foramen was seen in 32.7% of skulls and bilateral in 55.2%. In the same study, it was shown that the prevalence of parietal foramen was observed in 62.9% of skulls as single, 6.9% as double, 1.7% as triple, and 28.4% as not seen. Furthermore, they mentioned multiple parietal foramina in 13.8% of skulls. The average value of the distance between the sagittal suture and parietal foramen was 6.7 ± 2.9 mm on the right side and 6.8 ± 2.8 mm on the left side.^[4]^ In another study conducted in Telangana, the parietal foramen was detected in 64 of the 90 parietal bones. The parietal foramen was found in 10 skulls on the right side and 7 skulls on the left side. Bilaterally, the parietal foramen was observed in 23 skulls.^[19]^ Out of the 74 skulls, unilateral parietal foramen was observed in 20.3% of skulls and bilateral in 35.1% in a study performed in Saudi Arabia.^[20]^ And the mean distance of the parietal foramen from the sagittal suture were found as 9.68 ± 0.30 mm in right unilateral distribution while the mean distance of the parietal foramen from the sagittal suture were found as 13.77 ± 0.33 mm in left unilateral distribution in this study.^[20]^ In a study conducted in Brazil, the prevalence of the parietal foramen was 84.3%. Moreover the mean distance between parietal foramen and sagittal suture were reported as 7.1 ± 2.5 mm for male and 7.4 ± 2.7 mm for female in this study.^[21]^ In terms of the distance between the sagittal suture and the parietal foramen, the current study findings were similar to those of the abovementioned studies. A comparison of the study results with the data in the literature is shown in Table 2.

**Table 2:** Comparison of the study results with previous studies in terms of the prevalence of the parietal foramen.

Author	Region	Unilateral (%)	Bilateral (%)	Midsagittal (%)	Absent (%)	Present (%)
Berge 2001^[18]^	USA	30	49	–	20	79
Carolineberry 1967^[5]^	Egypt	–	–	–	55.8	44.2
	Nigeria	–	–	–	40.8	59.2
	Palestine (Lachish)	–	–	–	64.8	35.2
	Palestine (Modern)	–	–	–	77.8	22.2
	India	–	–	–	50.0	50.0
	Burma	–	–	–	50.0	50.0
	North America	–	–	–	38.0	62.0
	South America	–	–	–	47.0	53.0
Gagmei 2018^[9]^	India	29.2	62.5	–	–	–
Halagatti 2018^[3]^	India	–	–	–	24.4	75.6
Murlimanju 2015^[4]^	India	32.7	55.2		28.5	71.5
Singh 2011^[6]^	India	29	15	–	–	–
Salama 2023^[20]^	Saudi Arabia	20.3	35.1	12.2	9.5	90.5
Keskil 2003^[7]^	Turkey	31	27	5	37	63
Türk 2015^[22]^	Turkey				57.15	42.85
Present study	Turkey	25	27.5	0	47.5	52.5

When studies conducted in Turkey were focused, there was limited research on the parietal foramen. Keskil et al reported a parietal foramen at a rate of 63% and pointed out that the unilateral parietal foramen was more common (31%) than the bilateral parietal foramen (27%). They also reported a parietal foramen on the sagittal suture (5%).^[7]^ In another Turkish study, the prevalence was 42.85% on both sides, 51.78% on the right side, and 33.92% on the left side. Moreover, the mean distance of the parietal foramen to the midsagittal line was found as 1.49 ± 0.46 mm in total, 1.52 ± 0.51 mm on the right side, and 1.43 ± 0.36 mm on the left side.^[22]^ In another study the presence of parietal foramen was observed as 74.6% on right side and 74.7% on left side in study which performed with Turkish dry skulls. In this study the mean distance between parietal foramen and sagittal suture were found as 12.87 ± 1.25 mm on the left side and 11.95 ± 1.21 mm on the right side.^[23]^

Emissary veins provide a connection between the scalp veins and the venous sinuses of the brain. The parietal foramen is located posterior to the parietal bone and provides transmission way for the emissary veins. It plays an important role in the regulation of intracranial pressure and brain temperature by modifying the direction of blood flow through the emissary veins passing through the parietal foramen. Furthermore, the presence or absence of the parietal foramen is highly variational. Knowledge of the variations of the parietal foramen and it is relationships is extremely important to prevent emissery vein rupture during surgical operation.^[21,23]^

The limitation of this study is that the gender of the skulls used in the study is unknown. It is thought that the results of the study will shed light on forthcoming studies on larger samples with known gender skulls. As previously mentioned, there have been very few studies on the Turkish population; hence, further studies are required. Knowing the localization of the parietal foramen and its number and relationship with the sagittal suture aids in understanding the relationship between the dural venous sinuses and scalp veins. Data on these variations may also provide guidance for detecting congenital anomalies. Parietal foramen is thought to be associated with congenital anomalies such as cerebral venous anomalies, irregular suture fusion and sagittal suture deviation. Knowledge of the variations in morphometric characteristics of this region will guide the radiologist to diagnose lesions in the parietal bone. We believe that the results of this study will provide important morphometric data for anatomists and clinicians.

## Acknowledgments

The authors thank Ahmet Hilmi Yücel, Prof Dr, for her support, who passed away while the investigation was ongoing. This study was presented as a poster at the 20th National Anatomy Congress.

## Author contributions

**Conceptualization:** Nazire Kilic Safak, Ozkan Oguz.

**Data curation:** Nazire Kilic Safak.

**Investigation:** Nazire Kilic Safak, Zekiye Karaca Bozdag, Ayca Pamukcu.

**Methodology:** Nazire Kilic Safak, Zekiye Karaca Bozdag.

**Resources:** Zekiye Karaca Bozdag.

**Software:** Zekiye Karaca Bozdag, Ayca Pamukcu.

**Supervision:** Nazire Kilic Safak, Ozkan Oguz.

**Visualization:** Nazire Kilic Safak.

**Writing – original draft:** Nazire Kilic Safak, Zekiye Karaca Bozdag, Ayca Pamukcu.

**Writing – review & editing:** Nazire Kilic Safak.
